# Frequency of Hypoglycemia in Cirrhotic Patients Undergoing Endoscopy

**DOI:** 10.7759/cureus.62920

**Published:** 2024-06-22

**Authors:** Dilaram Khan, Inayat Ullah, Muhammad Yasir

**Affiliations:** 1 Gastroenetrology, Lady Reading Hospital Peshawar, Peshawar, PAK; 2 General Medicine, Lady Reading Hospital Peshawar, Peshawar, PAK

**Keywords:** child pugh class, hepatic encephalopathy, hepatocellular carcinoma, hypoglycemia, liver cirrhosis

## Abstract

Background: Liver cirrhosis is the replacement of normal liver parenchyma by fibrous tissue and nodularity. Cirrhosis liver has a negative effect on the body’s ability to regulate blood glucose levels because the liver cannot release the amount of glucose it would ultimately cirrhotic patients are at risk of hypoglycemia.

Objective: To determine the frequency of hypoglycemia in cirrhotic patients just before endoscopy after being nil per mouth (NPO) for 6 hours.

Methodology: This cross-sectional study was done at the Department of Gastroenterology Lady Reading Hospital, Peshawar from 1st April 2023 to 30th September 2023. Patients aged > 20 years of both genders, having Child-Pugh class C cirrhosis, and undergoing upper gastrointestinal (GI) endoscopy were enrolled in the study while patients with a history of diabetes using oral or parenteral hypoglycemic medications, patients taking steroids, patients with hepatocellular carcinoma and patients with hepatic encephalopathy were excluded. Diagnosis of liver cirrhosis was based on clinical examination, serum albumin level, and prothrombin time followed by characteristic findings on ultrasound. Serum glucose level was determined in the blood sample of the patient from the hospital laboratory.

Results: One hundred and ninety-six patients were enrolled including 130 (67.4%) males and 66 (32.6%) females. Age of the patients ranged from 20 to 60 years. The mean age of the participants was 46.68±10.239 years. Hypoglycemia was found in 48% of patients with liver cirrhosis. A significant association of hypoglycemia was found with disease duration and Child-Pugh class.

Conclusion: Hypoglycemia is a frequent finding in patients with liver cirrhosis and needs urgent management to prevent complications. Prolonged illness and patients with Child-Pugh class C cirrhosis are more likely to have hypoglycemia.

## Introduction

Liver cirrhosis is defined as the replacement of the normal liver parenchyma by dense fibrous tissue surrounding diffuse nodular regeneration with distortion of vascular meshwork in the liver. Causes of liver cirrhosis include viral hepatitis, excessive alcohol intake, non-alcoholic fatty liver disease, metabolic disorders, drug-induced hepatitis, inflammatory disorder, and cryptogenic. The most common cause is chronic viral hepatitis [1]. Worldwide mortality related to liver cirrhosis is approximately 1.6 million every year. The majority of the deaths related to cirrhosis of the liver are attributed to complications of cirrhosis including hepatic encephalopathy, hepatorenal syndrome, variceal bleeding, and liver cancer. The clinical course of liver cirrhosis is often intensified by electrolyte abnormality and hypoglycemia [2].

The liver is a vital organ of the body that plays a key role in glucose metabolism. It maintains and regulates the blood glucose principally through the glycogenolysis and gluconeogenesis. Liver dysfunction is correlated with poor blood glucose regulation [3]. The presence of the disturbance of liver metabolism, or liver cell damage may decrease the stability of the liver in regulating the blood glucose level. Hypoglycemia, defined as a blood glucose level of less than 70 mg per dl is not uncommon in cirrhotic patients [4]. A study by Qureshi and Rafiq reported hypoglycemia in 48.4% of patients with liver cirrhosis [5]. Another study by Mumtaz et al. reported a 33.3% prevalence of hypoglycemia in patients with liver cirrhosis [6]

The rationale of this study is that liver cirrhosis in Pakistan has an approximate prevalence of 27.5% [7] and hypoglycemia has been shown to increase mortality in cirrhotic patients [8]. So, the determination of the frequency of hypoglycemia in cirrhotic patients, particularly in our local population with less access to better health care, is of significance which will lead to the timely management of the hypoglycemia and ultimately decrease the mortality in these patients.

## Materials and methods

Study design

This cross-sectional study was conducted at the Department of Gastroenterology Lady Reading Hospital, Peshawar from 1st April 2023 to 30th September 2023 for six months using a non-probability consecutive sampling technique and after obtaining proper approval from the institutional review board of the Lady Reading Hospital No 1033/MTI/LRH dated 01/06/2023.

Inclusion criteria

Patients aged > 20 years of both genders, having Child-Pugh class C cirrhosis, and undergoing upper gastrointestinal (GI) endoscopy were enrolled in the study.

Exclusion criteria

 Patients with a history of diabetes mellitus using oral or parenteral hypoglycemic medications, patients taking steroids, patients with hepatocellular carcinoma, and patients with hepatic encephalopathy were excluded from this study.

Sample size

The sample size was 196 which was calculated as per the WHO sample size formula using the proportion (expected frequency of hypoglycemia) p=46.7% [9], margin of error= 7%, and confidence interval=95%.

Data collection procedure

All the 196 patients fulfilling the inclusion criteria were enrolled from the indoor and outdoor department of gastroenterology. Informed consent was taken from all study participants ensuring confidentiality and the fact that there is no risk involved while taking part in the study. Baseline information including age (years), gender, duration of disease, and cause of cirrhosis was noted.

Detailed history was taken from all patients followed by clinical examination. Signs of hypoglycemia such as dizziness, altered sensorium, and palpitation were noted. At our institution, patients undergoing upper GI endoscopy had to go through 6 hours of fasting as part of the upper GI endoscopy preparation protocol. In cirrhotic patients, the additional protocol is the determination of serum glucose level 30 minutes prior to endoscopy. For determination of serum glucose level, 5 cc blood was drawn from the patient in a blood sampling tube with a yellow cap and was sent immediately to the hospital laboratory for determination of blood glucose level. Hypoglycemia was noted as per operational definition (serum glucose level less than 70 mg/dl) in terms of frequency of hypoglycemia. Data was recorded by the researcher himself on a specially designed proforma

Data analysis

Data was analyzed by using SPSS (version 23). Frequencies and percentages were recorded for qualitative variables including gender, cause of cirrhosis, and presence of hypoglycemia. Mean ± standard deviation was computed for quantitative variables including age, and duration of disease. Effect modifiers like age, gender, disease duration, and cause of cirrhosis were controlled through stratification. Post-stratification chi-square test was applied. P-value ≤ 0.05 was considered statistically significant.

## Results

In this study, a total of 196 patients were registered including 130 (67.4%) males and 66 (32.6%) females. The age of the patients ranged from 20 to 60 years with a mean age of 46.68 ± 10.239 years; mean weight, mean height, and mean duration of disease are also shown in Table 1.

**Table 1:** Baseline demographics (n=196)

S.No	Variables	Mean ± SD
1	Patient age (years)	46.68 ± 10.239
2	Patient height (cm)	170.55 ± 7.883
3	Patient weight (kg)	72.07 ± 6.463
4	Disease duration (months)	38.71 ± 15.353

A total of 137 (69.7%) patients were aged more than 40 years, hepatitis C was the causative agent in 111 (69.7%) patients, 124 (63.4%) patients had the disease for more than 30 months, and hypoglycemia was found in 96 (48.0%) patients as shown in Table 2.

**Table 2:** Frequencies and percentages with respect to gender, age, cause of cirrhosis, disease duration, and hypoglycemia (n=196)

Variable	Sub-groups	Frequency	Percentage
Gender	Male	130	67.4
	Female	66	32.6
Age (years)	40 or below	59	30.3
	More than 40	137	69.7
Etiology of cirrhosis	Hepatitis B	85	43.4
	Hepatitis C	111	56.6
Duration of the cirrhosis (months)	30 or below	72	36.6
	More than 30	124	63.4
Hypoglycemia	Present	94	48.0
	Absent	102	52.0

Stratification of hypoglycemia according to gender, age, etiology of cirrhosis, and duration of cirrhosis is shown in Tables 3-6.

**Table 3:** Stratification of hypoglycemia according to gender (n=196)

Gender	Hypoglycemia		Total	p-value
	Present	Absent		0.596
Male	60 (46.2%)	70 (53.8%)	130(100%)	
Female	34 (51.5%)	32 (48.5%)	66 (100%)	
Total	94 (48.0%)	102 (52.0%)	196 (100%)	

**Table 4:** Stratification of hypoglycemia according to age (n=196)

Age (Years)	Hypoglycemia		Total	P-value
	Present	Absent		0.854
Forty or less	29 (49.1%)	30 (50.9%)	59 (100.0%)	
More than 40	65 (47.5%)	72 (52.5%)	137 (100.0%)	
Total	94 (48.0%)	102 (52.0%)	196 (100.0%)	

**Table 5:** Stratification of hypoglycemia according to etiology of cirrhosis (n=196)

Etiology of Cirrhosis	Hypoglycemia		Total	P-value
	Present	Absent		0.681
Hepatitis B (HBV)	40 (47.1%)	45 (52.9%)	85 (100.0%)	
Hepatitis C (HCV)	54 (48.6%)	57 (51.4%)	111 (100.0%)	
Total	94 (48.0%)	102 (52.0%)	196 (100.0%)	

**Table 6:** Stratification of hypoglycemia according to duration of cirrhosis (n=196)

Duration of Cirrhosis (Months)	Hypoglycemia		Total	P-value
	Present	Absent		< 0.001
Thirty or below	17 (23.4%)	55 (76.6%)	72 (100.0%)	
More than 30	77 (62.2%)	47 (37.8%)	124 (100.0%)	
Total	94 (48.0%)	102 (52.0%)	196 (100.0%)	

## Discussion

Cirrhosis liver is associated with several metabolic changes, catabolic in particular, leading to malnutrition in some cases [10]. The role of liver function in the management of the homeostasis of carbohydrates is important in considering the many physical and biochemical changes that occur in the liver in the presence of diabetes and considering how hepatic disease can alter the breakdown of glucose [11].

In this study, we evaluated the frequency of hypoglycemia in cirrhotic patients and observed that 94 (48%) of patients had hypoglycemic status results of this study are almost comparable to the study conducted by Tanveer et al. in Multan [12]. However, the results of our study are somewhat different from the study done by Singh et al. in Hyderabad where hypoglycemia was present in 67% of cirrhotic patients [13].

Generally, patients with liver cirrhosis have obvious glucose intolerance classified as hyperinsulinemia, hyper-glucagonemia, insulin resistance, and downregulation of insulin receptors. However, hyperinsulinemia may be caused by decreased liver regulation of insulin, hyper-glucagonemia is mainly because of elevated pancreatic discharge [14]. Multiple trials suggested that elevated blood glucose level in cirrhotic patients has a high risk of further destruction of the liver and a high mortality rate [3]. In this study, the ages of the patients ranged from 20 to 60 years. The mean age was 46.68 ± 10.239 years, the mean weight was 72.07 ± 6.463 kg, the mean height was 170.55 ± 7.883 cm, the mean duration of disease was 38.71 ± 15.353 months, and the mean serum albumin was 3.0680 ± 0.48281 g/dl. Similarly, Nouel et al. [15] noted hypoglycemia in 50% of their cirrhotic patients.

All these studies including our study stress the importance of checking blood glucose levels in cirrhotic patients after prolonged fasting to prevent mortality because hypoglycemia in cirrhotic patients is associated with a 30-day mortality of 30.2% [8].

Limitations of the study

The sample size was small and the study was conducted in a single center, so it may not represent the whole community of cirrhotic patients. Therefore, large-scale multi-center studies are needed to know about the exact frequency of hypoglycemia in cirrhosis liver patients, and ultimately necessary measures shall be planned to prevent hypoglycemia and the complications occurring because of hypoglycemia.

## Conclusions

Hypoglycemia is frequently found in cirrhotic patients after prolonged fasting especially those patients who are kept fasting for some endoscopic intervention or because of disease-associated anorexia and the majority of the studies stress the importance of checking blood glucose levels in cirrhotic patients after prolonged fasting to decrease the mortality because the hypoglycemia in cirrhotic patients is associated with high mortality and morbidity.
